# Plant- and Bacteria-Derived Compounds with Anti-*Philasterides dicentrarchi* Activity

**DOI:** 10.3390/pathogens11020267

**Published:** 2022-02-18

**Authors:** Rosa Ana Sueiro, José Manuel Leiro, Verónica Blanco-Abad, Jos Raaijmakers, Irene de Bruijn, Ron P. H. Dirks, Jesús Lamas

**Affiliations:** 1 Laboratory of Parasitology, Department of Microbiology and Parasitology, Institute of Research on Chemical and Biological Analysis, Campus Vida, University of Santiago de Compostela, E-15782 Santiago de Compostela, Spain; rosaana.sueiro@usc.es (R.A.S.); josemanuel.leiro@usc.es (J.M.L.); 2 Department of Functional Biology, Institute of Aquaculture, Campus Vida, University of Santiago de Compostela, E-15782 Santiago de Compostela, Spain; veronica.blanco.abad@usc.es; 3 Department of Microbial Ecology, Netherlands Institute of Ecology (NIOO-KNAW), 6708 PB Wageningen, The Netherlands; j.raaijmakers@nioo.knaw.nl (J.R.); idbruijn@koppert.nl (I.d.B.); 4 Future Genomics Technologies, Leiden BioScience Park, Sylviusweg 74, 2333 BE Leiden, The Netherlands; dirks@futuregenomics.tech

**Keywords:** *Philasterides dicentrarchi*, toxicity, natural compounds, *Pseudomonas*

## Abstract

*Philasterides dicentrarchi* is a scuticociliate that causes high mortalities in farmed fish. Although vaccination is an effective method to prevent scuticociliatosis caused by the homologous serotype, a universal vaccine has not been developed yet. Many compounds have been shown to be toxic to this ciliate species; moreover, most of them are toxic to aquatic life and cannot be used to prevent the disease. We have evaluated the toxicity to *P. dicentrarchi* of several compounds of natural origin to be used to reduce parasite levels in the seawater. Ciliates were exposed to several compound concentrations, and the mortality was determined at several incubation times. Tomatine, plumbagin and 2′,4′-dihydroxychalcone displayed the highest anticiliate activity, with a dose-dependent response. The effects of these compounds on the EPC cell line were also evaluated, finding that 2′,4′-dihydroxychalcone displayed the lowest toxicity to fish cells. At 7.54 μM, 2′,4′-dihydroxychalcone inhibited 50% parasite growth but only killed about 10% of EPC cells after 24 h incubation. Finally, we evaluated the toxicity of *Pseudomonas* H6 surfactant (PS) to *P. dicentrarchi*, finding that PS was toxic to the ciliate but showed lower toxicity to EPC cells. At a concentration of 7.8 μg/mL (LC_50_ for the ciliate after 3 h incubation), PS killed 14.9% of EPC cells. We conclude that 2′,4′-dihydroxychalcone, and PS could be used to reduce parasite levels in seawater, thus decreasing the risk of scuticociliatosis infection in cultured fish.

## 1. Introduction

The histiophagous scuticociliate *Philasterides dicentrarchi* (Ciliophora, Scuticociliatia) is the main aetiological agent of scuticociliatosis in cultured turbot [1,2,3] and is a serious threat to the aquaculture industry, causing high mortalities and significant economic losses [1,4,5]. *P. dicentrarchi* can induce severe systemic infections in turbot because of its ability to penetrate and spread throughout fish organs [6]. The endoparasitic location of this parasite makes treatment of scuticociliatosis difficult. At present, vaccination is the most effective method of preventing disease outbreaks [7,8,9]. However, experiments with different serotypes of this scuticociliate did not reveal any cross protection [10,11]. Until a universal vaccine is obtained, other methods must be developed to reduce the fish mortality associated with scuticociliatosis.

Many compounds have been reported to be toxic to *P. dicentrarchi* or to *Miamiensis avidus*, a scuticociliate parasite with many morphological similarities to the former. In an evaluation of the toxicity of 50 compounds, fourteen of these, including formalin, displayed antiparasitic activity [12]. A large group of compounds, including some newly synthesised agents, have also been found to show antiprotozoal activity [13,14,15]. However, many of the compounds evaluated, including formalin, are toxic to aquatic life and should not be used to reduce the number of ciliates in water or on the surface of infected fish. They also cannot be used to treat infected fish. In this respect, compounds with the antiprotozoal activity that also do not harm aquatic organisms are required. Products derived from plant extracts appear to be promising candidates for inclusion in novel treatments, complementary to vaccination and traditional drugs. Isolation, characterisation, and quantification of the bioactive compounds contained in plants are strongly recommended for use as new natural formulations for disease treatments in fish [16]. In this respect, we have identified several candidate compounds for developing antiscuticociliatosis drug treatments. Plant-derived polyphenolic compounds such as resveratrol, epigallocatechin gallate, mangiferin, and curcumin have been found to be effective against *P. dicentrarchi* [17,18,19]. Further studies with resveratrol have shown that inhibiting mitochondrial respiration is an important mechanism of anticiliate activity [20,21], but differences in the in vitro susceptibility to resveratrol observed in *P. dicentrarchi* isolated from turbot is also an important aspect to be considered in the potential use of this compound as an anticiliate agent [18].

*P. dicentrarchi* enters fish farms continuously through the feeder channels, and they increase in number in fish tanks as the temperature and organic matter levels rise, conditions that increase the risk of fish suffering from scuticociliatosis [22]. The aim of this study was to evaluate the toxicity to *P. dicentrarchi* of several compounds of natural origin, including some obtained from bacteria, for potential use to reduce the levels of the ciliate in the seawater or attached to the fish surface.

## 2. Results

### 2.1. Analysis of the Toxicity to P. dicentrarchi of Natural Compounds

We carried out initial screening tests to assess the toxicity to *P. dicentrarchi* of 26 compounds of natural origin at a concentration of 100 µM. The ciliates were incubated for 3 or 24 h in a culture medium containing the compounds (100 μM), and the percentage mortality (3 h) and growth inhibition (24 h) were determined (relative to the same measures in control groups). At the 3 h time point, four of the compounds, i.e., 2′,4′-dihydroxychalcone, 4-hexylresorcinol, plumbagin, and tomatine, killed more than 90% of the parasites, and after 24 h, these compounds had killed all the parasites (Table 1).

**Figure 1:**
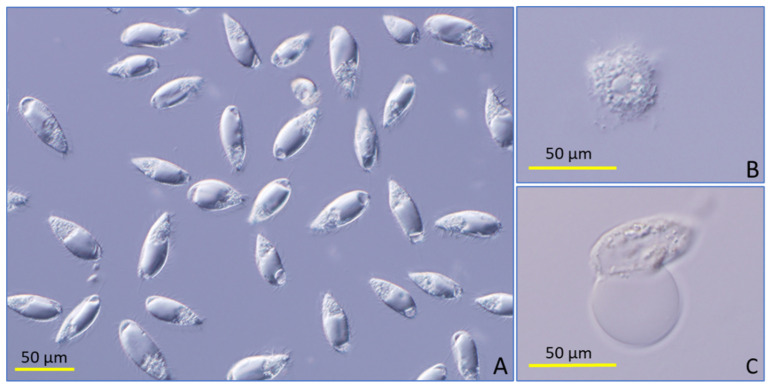
Nomarski microscopy images showing dead ciliates after incubation for 30 min with plumbagin (30 μM) (**A**), tomatine (10 μM) (**B**), or with 2′-4′-dihydroxychalcone (60 μM) (**C**).

Other compounds such as butyl 4-hydroxybenzoate, conessine, and piperine initially (after 3 h) displayed low toxicity but by 24 h had inhibited ciliate growth by more than 80%. These toxic compounds had a very different effect on *P. dicentrarchi*, i.e., tomatine caused very quick lysis of the ciliate, while plumbagin inhibited the ciliate movement, but it did not cause cell lysis for several hours (Figure 1).

**Figure 2:**
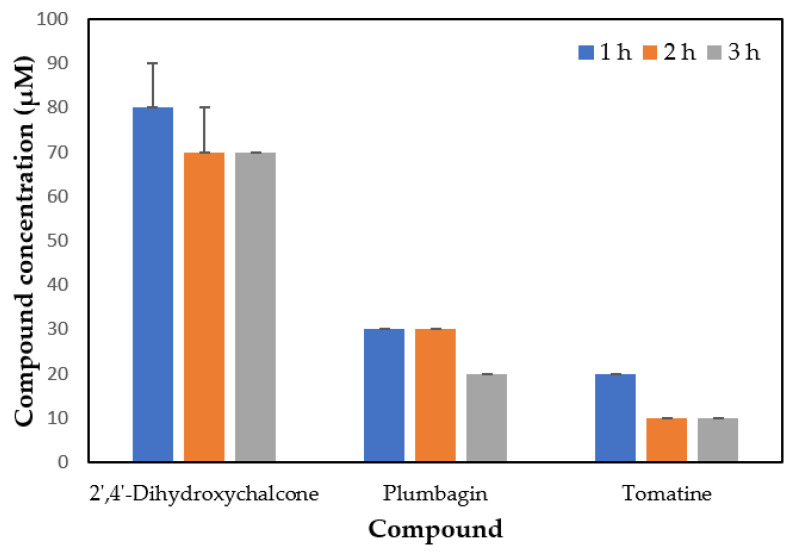
The lowest compound concentration that caused 100% ciliate mortality (LC_100_) after incubation of ciliates maintained in seawater with the compounds for 1, 2, or 3 h. Values are means ± SD of three replicates.

We then determined which compounds were the most effective against the ciliate cultured in seawater to be used to decrease parasite levels in the fish farm water tanks. The parasites were exposed to several concentrations of the seven compounds (0–100 μM, in 10 μM intervals) in seawater for 1, 2, or 3 h and the lowest dose of the selected compounds resulting in 100% ciliate mortality (LC_100_) was assessed. The toxicity of the compounds tested increased when parasites were cultured in seawater in comparison with ciliates cultured in a culture medium. However, only three of them killed all the ciliates at a concentration lower than 100 μM. Tomatine, plumbagin, and 2′,4′-dihydroxychalcone showed an LC_100_ of 10, 30, and 70 μM, respectively, after 3 h incubation with the ciliates (Figure 2).

**Figure 3:**
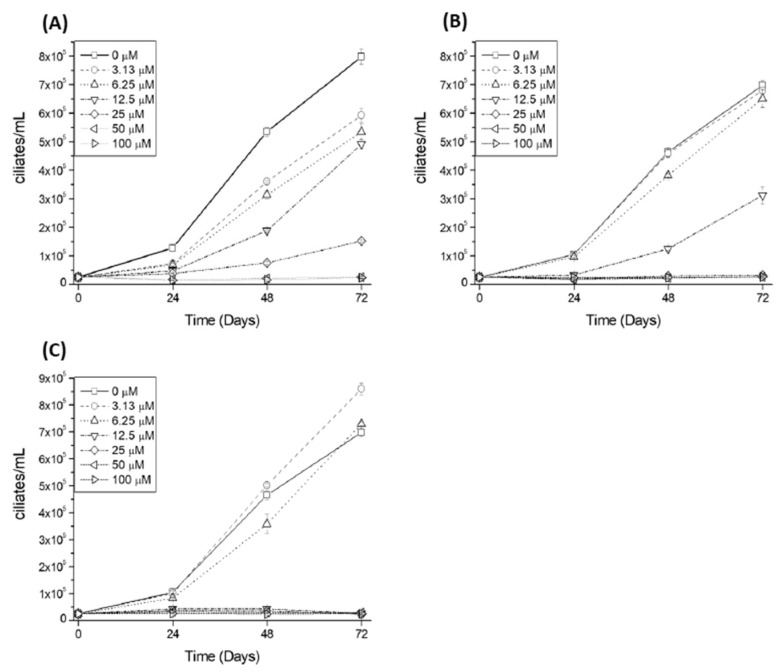
Antiparasitic effects of plumbagin (**A**), tomatine (**B**), and 2′-4′-dihydroxychalcone (**C**). The growth rate of *Philasterides dicentrarchi* incubated in a culture medium (control) or a medium containing different concentrations of the chemical compound (100–3.13 µM). The data shown are mean number ± SD (*n* = 3) of ciliates per mL after 0, 24, 48, and 72 h of incubation.

Ciliate growth in the presence of various concentrations of 2′,4′-dihydroxychalcone, tomatine, and plumbagin was evaluated at 0, 24, 48, and 72 h, and dose-dependent inhibition was observed at concentrations lower than 100 μM (Figure 3).

We then determined the compound concentration that inhibits 50% of ciliate growth (GI_50_), in comparison with the control group, finding that the GI_50_ for 2′-4′-dihydroxychalcone, tomatine, and plumbagin was of 7.54, 10.14, and 12.89 μM, respectively, after 24 h incubation (Table 2).

**Figure 4:**
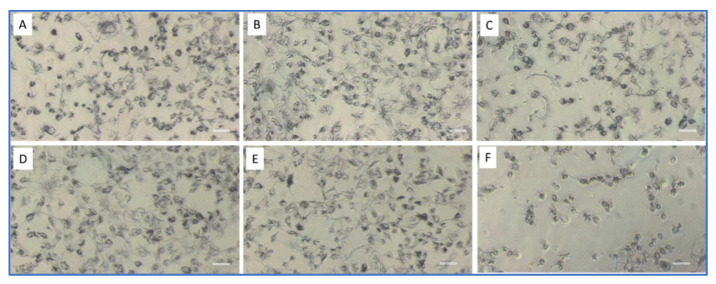
Photomicrographs showing the results of the MTT viability test of EPC cells incubated for 24 h with: (**A**) MEM, (**B**) 12.9 μM DMSO, (**C**) 7.54 μM 2′,4′ –dihydroxychalcone, (**D**) 12.9 μM plumbagin, (**E**) 10.14 μM tomatine and (**F**) 7.8 μg/mL H6 surfactant (PS). Bar = 20 μm.

Although these compounds can be obtained directly from plants, they may be toxic to plants or animals at the concentration used to kill the parasite. To test their toxicity on fish cells, we determined the effects of these compounds (used at a concentration that inhibits 50% of ciliate growth) on EPC cells, using the MTT assay as a test of their toxicity to fish cells. 2′,4′-dihydroxychalcone, which was highly toxic to *P. dicentrarchi*, displayed the lowest toxicity to fish cells, and at a concentration of 7.54 μM killed only about 10% of EPC cells (Table 3). By contrast, plumbagin and tomatine were more toxic to EPC cells, killing 26 and 43% of EPC cells, respectively, after incubation for 24 h (Table 3, Figure 4).

### 2.2. Effect of Pseudomonas H6 Surfactant on P. dicentrarchi

The toxicity of the *Pseudomonas* H6 surfactant (PS) to *P. dicentrarchi* was also investigated. This compound was found to be toxic for other ciliates that are also parasites of cultured fish. The ciliates were cultured in seawater and exposed to several concentrations of PS for 1, 2, and 3 h, yielding a lethal concentration 50 (LC_50_) values of 16.90, 9.0, and 7.8 μg/mL, respectively. Incubation of the ciliate with several concentrations of PS revealed a dose-dependent response, with the death of all the parasites exposed to 50 μg/mL of PS after 3 h. Evaluation of the toxicity of the compounds to EPC cells showed that PS was much less toxic to the cell line than to the parasite. At a concentration of 7.8 μg/mL (LC_50_ for the ciliate after 3 h incubation), PS killed 14.9 and 35.7% of EPC cells at 3 and 24 h incubation (Figure 4).

## 3. Discussion

*P. dicentrarchi* is an amphizoic ciliate that causes scuticociliatosis in many cultured fish species, especially flatfish, which can result in high mortalities and economic losses in fish farm systems. This ciliate species can feed on bacteria, microalgae, or other ciliates and can proliferate actively in seawater containing abundant organic matter. An increase in the number of ciliates in water probably increases the risk of scuticociliatosis at fish farms. In this respect, the use of methods to reduce the risk of scuticociliatosis by decreasing the number of ciliates in water or the development of treatments to reduce mortalities in infected fish may be very important in order to minimise economic losses. We tested the toxicity to *P. dicentrarchi* of 26 compounds of natural origin. The compounds found to be highly toxic to *P. dicentrarchi* included alkaloids (tomatine, connesine), quinoids (plumbagin), and polyphenols of the flavonoid family (2′-4′-dihydroxychalcone). Tomatine, plumbagin, and 2′-4′-dihydroxychalcone displayed the highest toxicity to the parasite. Tomatine is a mixture of two glycoalkaloids, α-tomatine and dehydroxytomatine, which are known to be present in both tomato leaves and fruits, and α-tomatine is particularly abundant in green tomatoes [23]. Tomatine has been reported to be involved in plant defence against bacteria, fungi, or viruses [24], and it has been shown to have antiprotozoal activity [25]. This compound has also been reported to have antiparasitic activity, inhibiting *Amyloodinium ocellatum* dinospore motility at doses in the range of 6.25–50 μg/mL (0.006–0.05 mM) during the incubation period (24 h) [26]. Similarly, tomatine (at 0.1 mM) also inhibited the radial growth of *Saprolegnia delica* and *S. parasitica* [26]. In the case of *P. dicentrarchi*, the tested compounds showed higher toxicity in seawater than in the culture medium. In seawater, tomatine at 10 μΜ killed the whole ciliate population in 3 h. However, this concentration only inhibited 50% ciliate growth after 24 h incubation in a culture medium. At the same concentration, tomatine was toxic for EPC cells, killing 43% of the cell population, suggesting that this compound may be toxic to fish. However, other studies have reported that administration of tomatine in the diet at doses from 100 to 2000 parts per million (ppm) for four weeks did not have toxic effects in rainbow trout [27]. The toxicity of tomatine for *P. dicentrarchi* and EPC cells may be related to permeabilisation of the plasma membrane, as previously observed with this and other saponins [28]. However, the toxic effect may be less important in vivo, and tests must be carried out to evaluate the toxicity to farmed fish susceptible to suffering scuticociliatosis. If there is no toxicity to fish at the concentration of 10 μM, tomatine is a very promising compound to control the *P. dicentrarchi* population in fish farms.

Plumbagin, which exhibits potent antibacterial and antifungal activities [29], has also been found to be toxic to other fish parasites inhibiting the growth of *Saprolegnia delica* at a dose of 0.1 mM (18.82 μg/mL) [26] or causing complete death of *Gyrodactylus kobayashii* within 45 min after exposure to 2.0 μg/mL [30]. In the present study, we found that plumbagin is toxic to EPC cells at a concentration of 12.89 μM (2.42 μg/mL), which kills 50% of the parasites and 26% of EPC cells. Plumbagin is toxic to goldfish at concentrations higher than 0.5 mg/L [30]. Assuming that it may also be toxic to other fish species at a similar concentration, it should not be used to reduce the levels of ciliates in tanks of seawater containing fish.

The other compound, 2′-4′-dihydroxychalcone, showed high toxicity for *P. dicentrachi*, but low toxicity for the EPC fish cell line. 2′,4′-dihydroxychalcone is a chalcone that belongs to the flavonoid family with two phenyl rings that it is present in plants, mainly as petal pigments, but have also been found in the heartwood, bark, leaf, fruit, and root [31]. This compound has been found to have antibacterial [32], antifungal [26], and antiparasitic activities [26,33]. Several polyphenols such as curcumin [19] or resveratrol [18], both with two phenyl groups, were reported to be toxic for *P. dicentrarchi*, although they showed lower toxicity than 2′,4′-dihydroxychalcone. While we did not establish why this compound is toxic for *P. dicentrarchi*, resveratrol, which showed a similar structure, has been found to affect several mitochondrial activities in the ciliate [20,21,34]. Although the toxicity of 2′,4′-dihydroxychalcone to fish needs to be evaluated, this compound is a highly promising drug to be used against *P. dicentrachi* in seawater to decrease ciliate levels. In addition, due to the low toxicity for fish cells, it is also a good candidate to be tested in the feed as a treatment against scutociliatosis.

In the search for other compounds that could be used to reduce *P. dicentrarchi* levels in seawater, we found that the *Pseudomonas* H6 surfactant was also highly toxic to *P. dicentrarchi*. This surfactant has been found to be toxic to other fish pathogens, including fungi, ciliates, and amoeba. Thus, it is toxic to the ciliate parasite *Ichthyophthirius multifiliis*, being 100% effective against theronts and tomocysts at concentrations of 10 and 13 μg/mL, respectively, after 60 min incubation; however, tomonts were only susceptible at concentrations above 100 μg/mL [35]. At concentrations higher than 250 µg/mL PS was toxic to amoebae that cause gill infections [36] and to fungi, almost completely inhibiting *Saprolegnia* hyphal growth at 100 μg/mL [37]. In the case of *P. dicentrachi*, PS killed 100% of the ciliates at 50–100 μg/mL after 1 h incubation, which is not very different from the values obtained for the other pathogens. We have also evaluated the toxicity of PS to the fish EPC cell line, finding that PS was far more toxic to *P. dicentrachi* than to the cell line. These results suggest that PS could be used to reduce the levels of *P. dicentrarchi* in seawater to reduce the risk of scuticociliatosis in fish farms.

The present study findings enabled us to conclude that some natural products such 2′,4′-dihydroxychalcone are toxic to *P. dicentrarchi* but less toxic to fish cells, suggesting that they could possibly be used to reduce parasite levels in water to prevent infections and could even be tested for inclusion in foodstuff to control infections. We also conclude that the *Pseudomonas* H6 surfactant is toxic to *P. dicentrarchi*, and it could also be used to reduce ciliate levels in water and prevent or reduce fish mortalities during outbreaks of scuticociliatosis.

## 4. Materials and Methods

### 4.1. Ciliate Culture

*P. dicentrarchi* (isolate I_1_), obtained from an outbreak of scuticociliatosis in cultured turbot in Galicia [1], was routinely cultured at 18 °C in complete sterile L15 medium (Leibovitz, PAA Laboratories GmbH, 1% salinity, pH 7.2) containing 90 mg/L each of adenosine, cytidine, and uridine, 150 mg/L of guanosine, 5 g/L of glucose, 400 mg/L of L-α-phosphatidylcholine, 200 mg/L of Tween 80, 10% of heat-inactivated foetal bovine serum (FBS), and 10 mL/L of 100X antibiotic–antimycotic solution (100 units/mL of penicillin G, 0.1 mg/mL of streptomycin sulphate and 0.25 mg/mL of amphotericin B), as previously described [38]. Alternatively, ciliates were cultured in seawater with autoclaved marine bacteria (10^6^ cells/mL) supplied as food at 21 °C. The ciliates were cultured under normoxic conditions in tissue culture flasks fitted with vented caps that enabled aeration of the culture medium.

### 4.2. Culture of the Epithelioma Papulosum Cyprini (EPC) Cell Line

We used the Epithelioma Papulosum Cyprini (EPC) cell line, acquired from the American Type Culture Collection (ATCC^®^, Manassas, VA, USA), as a control for toxicity. The cell line, kindly provided by Doctors Pereira-Dopazo and Bandin from the Aquaculture Institute of the University of Santiago de Coompostela, was cultured in MEM medium equilibrated with Hanks’ balanced salts (Gibco, Thermo Fischer Sci., Waltham, MA, USA) supplemented with 10% foetal bovine serum and 10 mL/L of 100X antibiotic–antimycotic solution. Cells were maintained subconfluent at 25 °C in a cell culture incubator and used when the confluence reached 75%.

### 4.3. Determination of Anticiliate Activity

We tested the toxicity to *P. dicentrarchi* of 26 compounds of natural origin (Table 1). The compounds were prepared in 10 mM stock solutions in dimethyl sulfoxide (DMSO) and maintained at −20 °C until use. In addition, the *Pseudomonas* H6 lipopeptide surfactant (PS) was prepared, lyophilised and stored at −20 °C [37]. A stock solution of PS was prepared in distilled water (1000 ppm) and diluted in culture medium or seawater at the final concentrations used in each assay and sterile filtered (through 0.2 µm filters) before being added to the parasites or EPC cells.

The anticiliate activity was assayed as previously described [12], with minor modifications [17]. Ciliates were concentrated by centrifugation at 650× *g* for 5 min and resuspended in a medium at the concentration required for the assays. All experiments were carried out in triplicate. Wells containing ciliates in a culture medium or seawater without the test compounds were assayed as negative controls. To rule out any possible effects of the solvent (DMSO), triplicate wells with a culture medium or seawater containing the highest concentration of DMSO used (up to 1%) were also included. The highest dose of solvent used (1%) had no effect on ciliate growth or viability. In these assays, the compounds were diluted in 1 mL of culture medium or seawater and added to the wells of 24-well sterile culture plates containing 2.5 × 10^4^ ciliates per well, at final concentrations of 3.23, 6.25, 12.5, 25, 50, and 100 µM in culture medium. The effects of the compounds on the motility and morphology of the ciliates were monitored periodically (at 30 min, 1, 2, 3, 24, and 48 h) under an inverted optical microscope. Ciliates were considered dead when they lysed and were not motile. The results obtained are expressed as percentage mortality for each experimental group. To estimate the total number of ciliates per well, the ciliates were inactivated by adding 0.25% glutaraldehyde and counted in a haemocytometer under an optical microscope [12]. The lowest dose of the selected compounds resulting in 100% ciliate mortality (LC_100_) was assessed at 1, 2, and 3 h after incubation of parasites with sample dilutions (from 0 to 100 μg/mL, in 10 μg intervals). The concentration that inhibited 50% of ciliate growth (GI_50_), in comparison with the control group, was calculated with Excel software (Microsoft Office; Microsoft, Redmond, WA, USA) by plotting the dose-response data (log dose-per cent of growth inhibition) and applying linear regression (y = mx + b) for data fitting. The values were then calculated from the equation log GI_50_ = (50 − b)/m, where m is the slope y_1_ − y_2_/x_1_ − x_2,_ and b is the intercept of the line.

### 4.4. Assessment of the Toxicity of Compounds on EPC Cell Line

Cultured EPC cells (1 × 10^5^ cells/well) were incubated in 100 µL of MEM medium with specific concentrations of each compound tested (the LC50 value obtained for each compound in the anticiliate assay on *P. dicentrarchi*) in a 96-well sterile culture plate (Corning) for 3 and 24 h at 25 °C and 5% CO_2_. Cell viability was then estimated by the MTT (3-(4,5-dimethylthiazol-2-yl)-2,5-diphenyl tetrazolium bromide) assay, as previously described [39], with minor modifications. Briefly, 100 μL of the MTT solution (1 mg/mL in phosphate-buffered saline; pH 7.2) was added to each well, and plates were incubated at 25 °C for 4 h. The yellow tetrazolium salt of MTT is reduced by mitochondrial dehydrogenases in metabolically active cells to form insoluble purple formazan crystals, which are then dissolved by the addition of DMSO (50 μL/well). Prior to the addition of MTT or DMSO, the plates were washed, and the supernatant was removed. The absorbance was measured at 540 nm in a microplate reader (Multiskan™, Thermo Fischer Sci., Waltham, MA, USA), and the relative cell viability was expressed as the mean percentage of viable cells relative to untreated cells.

In the viability assay, the possible effect of DMSO was evaluated for each compound by the inclusion of three replicate wells with MEM medium and the same DMSO concentration as in the medium. The highest dose of solvent used did not affect cell proliferation or viability. Control cells with only MEM were also included in the assay.

### 4.5. Statistics

The results are expressed as means ± standard deviation (SD), and statistical significance was assessed by one-way ANOVA followed by Tukey–Kramer tests for multiple comparisons.

## Figures and Tables

**Table 1:** Per cent ciliate mortality and growth inhibition after incubation of ciliates maintained in cultured medium with several compounds of natural origin (100 μM) for 3 or 24 h, respectively. Values are means ± SD of three replicates. Suppliers: SC (Santa Cruz, Heidelberg, Germany), S-A (Sigma-Aldrich, Amsterdam, The Netherlands), SB (Sanbio, Uden, The Netherlands). Ref. number: reference number.

Compound	Supplier and Ref. Number	Ciliate Mortality 3 h (%)	Growth Inhibition 24 h (%)
2′,4′-dihydroxychalcone	SC (sc-266263)	99 ± 0.71	100
4-hexylresorcinol	S-A (209465)	95.3 ± 4.05	100
7-hydroxyflavone	S-A (H4530)	10 ± 1.52	67.39 ± 2.4
Benzoic acid	S-A (33045)	1.64 ± 0.56	17.39 ± 1.2
Biotin	S-A (B4501)	0	36.96 ± 0.72
Butyl 4-hydroxybenzoate	S-A (54680)	52.88 ± 2.39	80.65 ± 2.42
Camphor (1r)	S-A (857300)	0	77.42 ± 2.7
Cedrol	S-A (W521418)	64.25 ± 3.7	66.67 ± 0.33
Conessine	SB (11700)	50.25 ± 4.5	84.42 ± 2.42
Coumarin	S-A (C4261)	8 ± 5.90	23.47 ± 4.12
Diallyl sulfide	S-A (W204218)	22.3 ± 5.1	38.76 ± 7.32
Esculetin	S-A (246573)	8.2 ± 5.32	15.31 ± 6.32
Eucalyptol	S-A (C80601)	17 ± 8.42	35.62 ± 4.21
Garlicin 80%	S-A (317691)	30.02 ± 0.9	42.47 ± 5.62
Harmalol hydrochloride dihydrate	S-A (H125-1G-A)	4.8 ± 1.12	40.08 ± 4.9
Monocrolatine	S-A (C2401)	6.61 ± 1.06	9.8 ± 2.11
Palmatine chloride	S-A (sc-205788)	6.8 ± 0.59	28.46 ± 4.25
Phenylethyl alcohol	S-A (W285811)	2.3 ± 1.4	30.14 ± 4.63
Piperine	S-A (P49007)	42.6 ± 9.1	89.04 ± 3.37
Plumbagin	SC (sc-253283)	100	100
Rosmarinic acid	S-A (536954)	27.12 ± 7.27	35 ± 5.51
Sclareolide	S-A (W379401)	62.64 ± 4.6	71.67 ± 6.92
Sodium lactate	S-A (71718)	13.76 ± 4.1	28.33 ± 3.93
Tomatine	SC (sc-296548)	93.33 ± 2.31	100
Umbelliferone	S-A (H24003)	2.9 ± 1.89	44.94 ± 7.23
Usnic acid	S-A (329967)	38.33 ± 5.52	68.3 ± 3.34

**Table 2:** The compound concentration that inhibited 50% of parasite growth (GI_50_) in a cultured medium at 24 or 48 h. Values are means ± SD of three replicates. Different superindices represent statistically significant differences among compounds of the same group (24 or 48 h) (*p* < 0.05).

Compound	GI_50_ 24 h (µM)	GI_50_ 48 h (µM)
2′-4′-Hydroxychalcone	7.54 ± 1.31	5.68 ± 0.16
4′-Hexylresorcinol	63.31 ± 3.61	40.80 ± 0.11
Butyl-4-hydroxybenzoate	43.92 ± 6.60	29.22 ± 0.35
Piperine	60.70 ± 6.31	43.97 ± 1.83
Plumbagin	12.89 ± 1.16	9.65 ± 0.67
Tomatine	10.14 ± 1.27	8.51 ± 0.20
Conessine	12.02 ± 0.80	11.21 ± 0.65

**Table 3:** Per cent EPC cell viability determined using the MTT assay after incubation for 3 or 24 h with the compounds used at the concentration that inhibit 50% ciliate growth (GI_50_) in 24 h. Values are means ± SD of three replicates. * *p* < 0.05.

Compound	GI_50_ (24 h)	EPC Cell Viability (%) Mean ± SD
2′,4′-dihydroxychalcone	7.54 μM	
3 h		92.04 ± 2.50
24 h		89.69 ± 1.40
Plumbagin	12.89 μM	
3 h		78.69 ± 1.40 *
24 h		74.16 ± 3.94 *
Tomatine	10.14 μM	
3 h		62.99 ± 6.82 *
24 h		57.63 ± 7.70 *

## Data Availability

Not applicable.
